# 

**Published:** 1871-12

**Authors:** 


### CONTENTS:

### ORIGINAL CONTRIBUTIONS.

Lecture xlii. — Erysipelas — Sporadic and Epidemic. By N. S. Davis, etc., 639
Bromide Potassium in Uræmic Convulsions. By E.A. Carpenter, M.D., 652
Cundurango. By E. Andrews, etc.... 656

### CLINICAL REPORTS.

Cases in Mercy Hospital—Gynæcological Wards. Prof. Byford ........... 661
Medical Wards. Prof. N. S. Davis... 664
Surgical Wards. Prof. Andrews ....... 666
Opthalmological Wards. Prof. Jones, 669

### SELECTIONS.

Report of the Committee of Amer. Med. Ass'n, etc. Read May 2, 1871 671
Report on Otology. By S. J. Jones, A.M., M.D., etc ........ 680
Recent Foreign Papers ........ 684
Researches on Inflammation and Suppuration ........ 684
Passage of Corpuscles Through the Blood-vessels ........ 686
Albuminuria in Variola ........ 687
Two Cases of Paralysis of the Third Nerve. By C. K. Fiske, M.D ........ 688
Extract of Conium in Inflammation of the Breast ........ 690
Poisons on Different Animals ........ 690
Infection—ready mode of preventing, 691
Explorations of Coast Survey Bureau, 691
The Removal of an Inverted Womb... 691
Malignant Small-Pox, Treated with Sulphur Fumigations, etc. By F. Hjaltelin, M.D., etc ........ 692
Intra-pericardial Aneurism of Aorta, 692
Wet Sheets in Diarrhœa ........ 693
Transplantion of Mucous Membrane, 694
A Sound Lodged in the Uterus ........ 694
Sesquichloride of Iron in Rheumatism 695
Compression Treatment of Aneurism. 696
Syphilitic Ulcers ........ 697
Fissure of the Anus ........ 697
Dysmenorrhœa ........ 697
Gangrene Treated by Turpentine ........ 697
Carbolic Acid as a Local Anæthetic... 697
Chloral and Cod Liver Oil ........ 698
Sulphuric Ether in Midwifery ........ 698
Bromide of Sodium ........ 698
Bisurphate of Lime as a Deodorizer... 699

### BOOK REVIEWS

Transactions of Amer. Med. Ass'n.... 699
Trans. Ohio State Med. Society ........ 704
Surgeon-General's Report, U.S.A. .... 705
Trans. Pennsylvania Med. Society...... 706
Principles and Practice of Medicine. By Henry Hartshorne, A.M., M.D., 707

### EDITORIAL.

Our French Exchanges ........ 707
Rush Med. College. Correction ........ 708
The Present and the next Volume...... 709
Chicago Medical Soeiety ........ 709
Illinois State Microscopical Society.... 709
Circular. State Med. Society ........ 711
Mortality, for October, 1871 ........ 712
Mortality, for November, 1871 ........ [illegible]

